# Impact of an enterprise controlled substance management system on labor and inventory costs

**DOI:** 10.1093/ajhp/zxae305

**Published:** 2024-10-26

**Authors:** Michael Mohundro, Thomas Greene, Cindy Moore, Jennifer Jones, Claudia Goldblatt, Heather Nelkin, Amanda Hays

**Affiliations:** Franciscan Missionaries of Lady Health System, Baton Rouge, LA, USA; Franciscan Missionaries of Lady Health System, Baton Rouge, LA, USA; Franciscan Missionaries of Lady Health System, Baton Rouge, LA, USA; Franciscan Missionaries of Lady Health System, Baton Rouge, LA, USA; Becton, Dickinson and Company, San Diego, CA, USA; Becton, Dickinson and Company, San Diego, CA, USA; Becton, Dickinson and Company, San Diego, CA, USA

**Keywords:** automatic dispensing cabinets, controlled substances, discrepancy, inventory management, PAR levels

## Abstract

**Purpose:**

To evaluate the impact of an enterprise controlled substance (ECS) management system with integration of analytics software on labor requirements and inventory cost within a health-system pharmacy.

**Methods:**

A prospective, pre-post observational study was designed to assess the impact of implementing a solution that connects disparate systems with the integration of analytics software. Three study modules were implemented over approximately 18 months. The intervention consisted of implementation of new CS vaults, a centralized server, an automated central medication inventory system, and inventory optimization analytics software. The number of transactions and time spent on CS reports were compared before and after implementation to determine labor and inventory efficiencies.

**Results:**

Both of the study facilities had a decrease in CS daily stockouts and an increase in inventory turns compared to baseline, while the total number of transactions (vends) at the central vault and decentralized dispensing cabinets increased. The addition of analytics allowed for establishment of informed changes to periodic automated replenishment levels. Additionally, both facilities saw a reduction in the number of expired medications, and there was subsequently a reduction in the total reverse distributor costs. Finally, both facilities had a reduction in the amount of time spent on manual tasks associated with reconciling and managing discrepancies.

**Conclusion:**

An inventory management system integrated with an advanced analytics tool provided a reduction in the time spent on receiving, storing, and reconciling CS records while the number of transactions increased. The ECS solution enhanced the visibility of the chain of custody while closing the loop between reporting and receiving inventory, eliminating or reducing the frequency of manual processes.

**Key Points**

- Despite an increase in the number of transactions at the central controlled substance vault and automated dispensing cabinets (ADCs) on the floor, there was a decrease in stockouts and removal of expired medications with automated inventory management.
- Less time was spent on inventory tasks such as receiving, stocking, and vending at the vault and ADCs; time saving was also observed for report comparisons and managing discrepancies with the new software.
- There is increased visibility of current inventory with establishment of periodic automated replenishment levels, which improved efficiency over manual ordering.

Healthcare organizations are responsible for developing a controlled substance (CS) diversion prevention program that complies with applicable laws and regulations at the state and federal levels.^1^ According to the Drug Enforcement Administration (DEA), registrants are responsible for ensuring the proper management of CSs throughout the medication use process.^2^ They are also responsible for utilizing technology as applicable to enhance surveillance and oversight as a means to help prevent and/or detect potential diversion.^1^ Over the past several years, there has been heightened diversion awareness and numerous healthcare organizations have come under scrutiny as a result of ineffective CS management, including cases involving regulatory bodies and law enforcement when the chain of custody was not maintained properly, allowing for drug diversion.^3,4^ Besides reputational damage and direct costs to healthcare systems, diversion threatens patient and healthcare worker safety and can lead to other unintended consequences.^5-8^

Pharmacy leadership has the responsibility to ensure end-to-end medication management, including ensuring ordering accuracy, inventory management, removing expired medications, and addressing missing waste and missing documentation.^9^ Historically, these tasks have been manual, labor intensive, and ultimately time consuming.^10^ Reconciling wholesaler invoice data is one example of a manual process, which can lead to gaps in oversight of purchasing and receiving workflows.

CS inventory management is a complex process with multiple steps in the chain of custody.^1^ Typical activities may include ordering, delivery to facilities, receipt upon arrival to the pharmacy, distribution to specific floor units, possible dispatch to other locations (ie, “sell-to” areas) within the same healthcare system, administration to patients, and possible disposal or return of unused medications. A goal of many organizations is to improve inventory management by ensuring that inventory turns occur frequently enough to reduce excess cost.

The 2022 American Society of Health-System Pharmacists (ASHP) guidelines on preventing diversion of CSs^1^ highlight the need to implement automated systems that support CS surveillance and auditing in high-risk areas such as the main pharmacy CS vault. Effective CS management can minimize labor and medication costs and enhance the opportunities for detection of diversion.^1,11,12^ This can be achieved through careful planning and the use of technology to support early detection and monitoring of the chain of custody. This study was designed to assess the impact of an enterprise CS (ECS) management system with integration of inventory analytics software on labor requirements and inventory cost.

## Methods

### Setting

This study was conducted at 2 hospitals (facility 1 and facility 2) in a 10-hospital nonprofit, academic health system located in the southeastern US. The health system also has multiple offsite locations, including ambulatory surgical centers, infusion centers, behavioral health facilities, and freestanding emergency departments. CSs are transferred by a sell-to process from the various pharmacy DEA registrants to offsite locations. Facility 1 is an 800-bed multispecialty level 1 trauma center and American Nurses Credentialing Center Magnet–designated facility with over 135 automated dispensing cabinets (ADCs). Facility 1 dispenses approximately 44,000 CSs per month across the facility. Facility 2 is a 321-bed level 3 trauma center with 91 ADCs and dispenses approximately 24,000 CSs per month across the facility.

While the facilities are in the same health system, workflows varied based on differences in practice site. Facility 1 had a designated CS technician whose duties included oversight and recordkeeping compliance for CSs. In addition, the CS technician received CSs into the vault and delivered CSs to the patient care units daily. Facility 2 had a designated pharmacist who provided oversight and recordkeeping compliance for CSs.

Ordering practices were also different for the 2 sites in the preimplementation period. Facility 1 utilized the DEA’s CSOS (Controlled Substance Ordering System) for daily ordering, while facility 2 used a manual process with paper DEA 222 forms for CS ordering weekly. The sites utilized the same wholesaler for CS ordering and the same 503B facilities for additional purchases of compounded and ready-to-use CSs. They also utilized the same reverse distributor; however, the pickup schedule was different for the facilities, with facility 1 scheduled monthly and facility 2 scheduling on an ad hoc basis. Additionally, the 2 locations used a common drug diversion analytics software application for proactive monitoring of unreconciled transactions and anomalous behaviors. The new ECS management system allows for coordinated efforts, with a high-level view that is less focused on individual hospitals. The responsibilities of DEA registrants can more effectively be met to manage the chain of custody for CSs when reports are not isolated to siloed CS vaults; an integrated delivery network (IDN) solution increases visibility of all CS transactions.^2^

### Study design and methodology

This study was designed as a prospective, single-enterprise, multicenter, institutional review board–exempt, pre-post observational study to assess the impact of an ECS solution on labor, inventory, costs, stockouts, and periodic automated replenishment (PAR) management. The ECS solution consists of the following technologies: CS vaults (BD Pyxis C^II^ Safe ES; BD, San Diego, CA), a centralized server (BD Pyxis ES 1.7), automated central medication inventory systems (BD Pyxis Logistics) utilized with the organization’s existing storage carousels (SencorpWhite Vertical Carousel Systems; SencorpWhite, Hyannis, MA), and inventory optimization analytics (IOA) software (BD HealthSight Inventory Optimization). The IOA software is a descriptive analytics solution intended to automate historically manual optimization processes, including PAR changes and withdrawals.^11^ The IOA solution consolidates visibility of inventory within the ADCs, carousels, and CS vaults.^11^ The CS safe on a centralized server integrates CS ordering, receiving, and inventory management. This solution allows an enterprise-level view when managing CSs rather than one that is focused on individual hospitals.

The preimplementation phase of the study lasted 30 days (March 31 to April 29, 2022), and the postimplementation phase took place over a 91-day period (July 5 to October 3, 2023). Between the pre- and postimplementation phases, the inventory management system was installed followed by a 91-day adoption period to allow for stabilization of a new workflow.

### Study endpoints

Data from ADCs and centralized CS vaults were analyzed for changes to inventory practices and time spent by staff on routine activity. The study was divided into 3 modules, each with unique objectives and endpoints. Definitions for transaction types are provided in eTable 1. The first module assessed inventory labor requirements by comparing the following metrics before and after ECS implementation: the number of CS vault and ADC transactions, the number of patient-specific withdrawals or “vends,” the number of vends to ADCs on the floor, stockouts, PAR level changes, the number of expired medications, and inventory turns. Inventory turns were captured over 39 days at baseline and for 91 days in the postimplementation period. The ECS solution was hypothesized to improve ordering accuracy; therefore, it was expected that the number of stockouts would decrease and CSs would be refilled at more appropriate times (without medications sitting for excessive lengths of time, risking expiration). Time spent dispensing CSs, receiving and stocking inventory, and reconciling discrepancies was monitored to measure labor requirements.

The second module examined direct costs tied to the ECS solution, including reverse distributor costs, costs of expired CSs, and costs of unused CSs. Inventory turns were assessed to determine the rate of stock replacement; low turnover rates indicate increased costs due to items being stored for long periods of time.^13,14^

Lastly, the third module assessed the perceptions of key pharmacy personnel about the CS management using a 5-point Likert scale. The survey was deployed to key personnel involved in the pharmacy operations related to CSs both before and after implementation. A total of 26 surveys were distributed in the preimplementation period, and 57 were distributed in the postimplementation period. The survey was sent to more pharmacy staff in the postimplementation period based on the new functionality of the CS vault software that connected additional solutions for ordering, receiving, and managing inventory.

### Data collection

A mixed-methods approach was taken for data collection utilizing manual audit logs (Microsoft Forms; Microsoft Corporation, Redmond, WA) and reports from the CS vault. Preimplementation data were collected before the CS vault went live in a production environment. Postimplementation data were collected retrospectively from the automated CS vault 91 days after the implementation period and prospectively with manually logged data. Where relevant, estimates from the postimplementation period were divided by a factor of 3.03 (91/30) and rounded to the nearest whole number to account for the different lengths of time for data collection during the pre- and postimplementation periods and to achieve comparability between data points. All data were deidentified.

### Statistical analysis

Descriptive statistics were calculated using Python Version 3.6+ (Python Software Foundation, Beaverton, OR) and Microsoft Excel for all endpoints before and after the intervention at the 2 facilities. Where relevant, Student’s *t* tests were used to test for statistical significance. An a priori level of significance was set at 0.05. All statistical analyses were conducted using SAS Version 9.4 (SAS Institute, Cary, NC). Perception survey results were obtained and analyzed using Qualtrics (Silver Lake, Seattle, WA) and Microsoft Excel.

## Results

### Module 1

**Figure 1.**
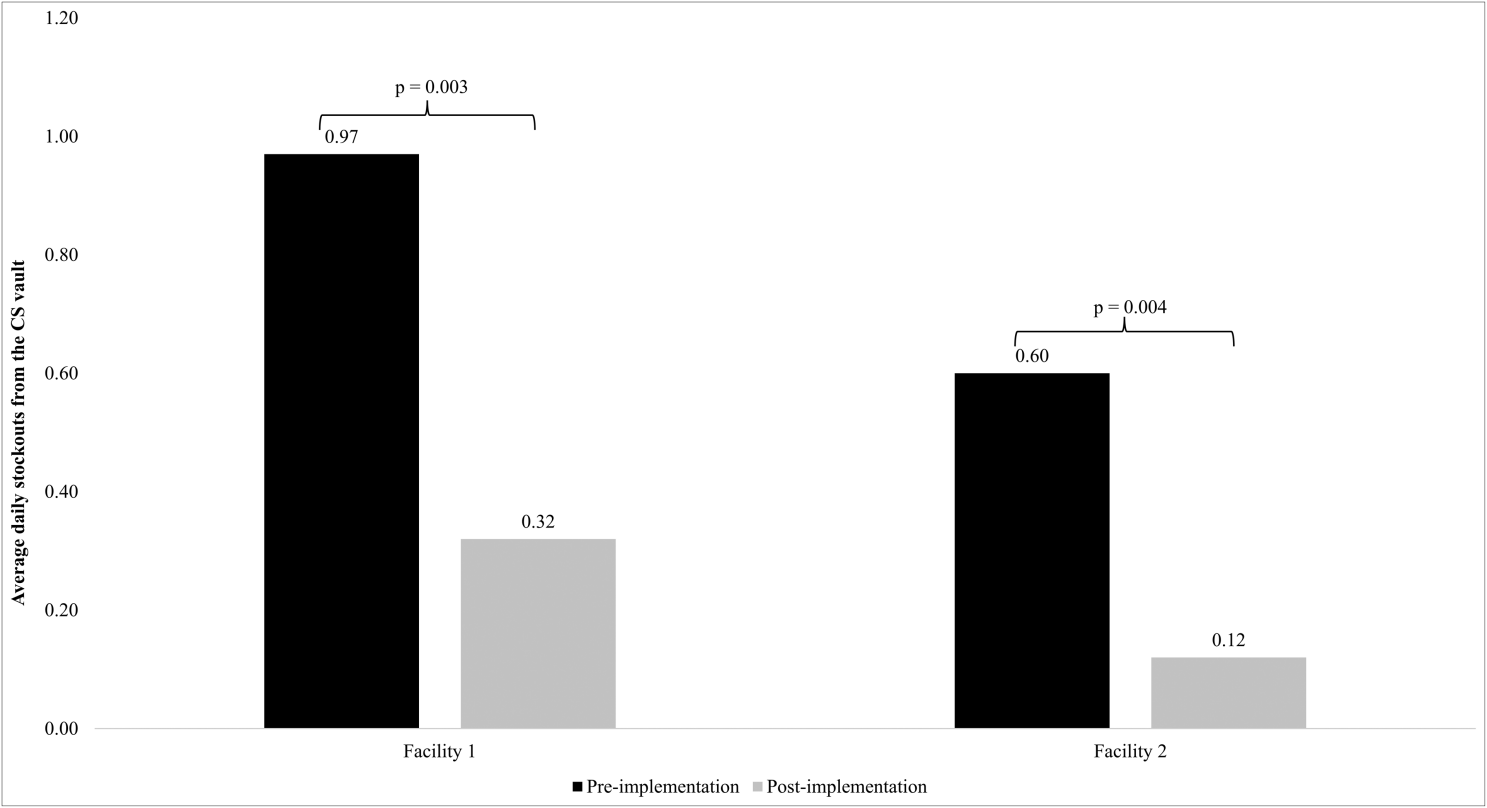
Change in average daily stockouts from the controlled substance (CS) vault.

***Inventory labor requirements*.** Both facilities had proportional increases in the total number of vends from the CS vault recorded, while the number of transactions involving expired medications decreased at both facilities (Table 1). The proportion of CS stockouts and the average number of daily stockouts from the vault decreased at both facility 1 (*P* = 0.003) and facility 2 (*P* = 0.004) (Figure 1).

**Table 1.** Controlled Substance Vends, Loss to Expiry, and Stockouts

Facility	CS vends from CS vault, No.^a^		Transactions with expired CS removed, No.^a^		CS stockout, %	
	Before	After	Before	After	Before	After
1	3,227	3,629	142	32	0.9	0.26
2	2,569	3,044	102	10	0.7	0.12

Abbreviation: CS, controlled substance.

^a^Data collection for the preimplementation period took place over 30 days (March 31 to April 29, 2022), while data collection for the postimplementation period took place over 91 days (July 5 to October 3, 2023). To achieve comparability between data points, estimates from the postimplementation period were divided by a factor of 3.03 (91/30) and rounded to the nearest whole number.

#### Inventory management

Transactions at the central CS vault were also reviewed to measure the impact of the new system, specifically, the time devoted to inventory tasks. The volume of transactions related to receiving and stocking decreased in the postimplementation period, and, in comparison to baseline, both facilities saw a reduction in the amount of time spent on receiving and stocking the vault (Table 2). The average time spent on these actions also decreased when measured per transaction.

**Table 2.** Receiving and Stocking of Controlled Substances in the CS Vault

Facility	Transactions for receiving and stocking CSs in the CS vault, No.^a^		Average time per transaction for receiving and stocking CSs in the CS vault, minutes	
	Before	After	Before	After
1	51	4	26.8	9.3
2	17	10	67.2	28.6

Abbreviation: CS, controlled substance.

^a^Data collection for the preimplementation period took place over 30 days (March 31 to April 29, 2022), while data collection for the postimplementation period took place over 91 days (July 5 to October 3, 2023). To achieve comparability between data points, estimates from the postimplementation period were divided by a factor of 3.03 (91/30) and rounded to the nearest whole number.

#### PAR levels and inventory turns

As the PAR inventory system did not include CSs before implementation of the ECS system, no PAR level changes were reported at baseline. This resulted in an increase in the average number of daily PAR level changes at facility 1 (*P* = 0.02) and facility 2 (*P* = 0.15). Both facilities had an increase in inventory turns for the CS vault in the postimplementation period (Table 3).

**Table 3.** PAR Level Changes and Inventory Turns in the CS Vault

Facility	Total PAR level changes in the CS vault, No.^a^		Average daily PAR level changes in the CS vault			Inventory turns^b^	
	Before	After	Before	After	*P* value^c^	Before	After
1	0	17	0	0.56	**0.002**	2.73	6.76
2	0	3	0	0.09	0.15	0.18	1.03

Abbreviations: CS, controlled substance; PAR, periodic automatic replenishment.

^a^Data collection for the preimplementation period took place over 30 days (March 31 to April 29, 2022), while data collection for the postimplementation period took place over 91 days (July 5 to October 3, 2023). To achieve comparability between data points, estimates from the postimplementation period were divided by a factor of 3.03 (91/30) and rounded to the nearest whole number.

^b^Inventory turns for the preimplementation period were calculated for a 39-day period using the following formula: total purchase cost/average daily inventory cost.

^c^Significant *P* values are shown in bold.

#### Reconciliation and discrepancy management

Analysis of CS vault records also showed that the average time spent per transaction by staff managing discrepancies and reconciling surveillance reports decreased at both facilities. Both facilities had a greater number of transactions logged for managing discrepancies in the postimplementation period but had fewer transactions for managing and reconciling surveillance reports (Table 4).

**Table 4.** Managing Discrepancies and Reconciling Surveillance Reports for the CS Vault

Facility	Transactions where discrepancies were created, No.^a^		Average time per transaction for managing discrepancies, minutes		Transactions for reconciling surveillance reports, No.^a^		Average time per transaction for reconciling surveillance reports, minutes	
	Before	After	Before	After	Before	After	Before	After
1	1	2	10	5.3	65	6	29.9	8.1
2	2	3	53.5	3.3	66	16	4.8	0.8

Abbreviation: CS, controlled substance.

^a^Data collection for the preimplementation period took place over 30 days (March 31 to April 29, 2022), while data collection for the postimplementation period took place over 91 days (July 5 to October 3, 2023). To achieve comparability between data points, estimates from the postimplementation period were divided by a factor of 3.03 (91/30) and rounded to the nearest whole number.

#### Vending transaction time

In a similar manner, while there was an increase in the number of vends from the vault to the ADCs on the floor during the postimplementation period, the average time spent performing each transaction decreased (Table 5). Total time spent dispensing patient-specific medications from the vault decreased at facility 1 but increased at facility 2. Facility 1 saw an increase in the volume of patient-specific dispenses but limited available space in ADCs. However, the average time per transaction for patient-specific dispenses was similar at the 2 facilities.

**Table 5.** Vending Controlled Substances From the CS Vault for Automated Dispensing Cabinets on the Floor

Facility	Patient-specific dispenses from CS vault, No.^a^		Total time for patient-specific dispensing from CS vault, minutes^a^		Average time per transaction for patient-specific dispensing from CS vault, minutes		Vends from CS vault for ADCs on the floor, No.^a^		Total time to vend items from CS vault for ADCs on the floor, minutes^a^		Average time per transaction to vend items from CS vault for ADCs on the floor, minutes	
	Before	After	Before	After	Before	After	Before	After	Before	After	Before	After
1	192	184	1,118	796	5.8	4.3	3,035	3,445	88,081	21,163	29	6.1
2	291	296	577	1,353	2.0	4.6	2,278	2,748	33,768	37,416	14.8	13.6

Abbreviations: ADC, automated dispensing cabinet; CS, controlled substance.

^a^Data collection for the preimplementation period took place over 30 days (March 31 to April 29, 2022), while data collection for the postimplementation period took place over 91 days (July 5 to October 3, 2023). To achieve comparability between data points, estimates from the postimplementation period were divided by a factor of 3.03 (91/30) and rounded to the nearest whole number.

### Module 2

***Inventory costs*.** At facility 1, reverse distributor costs could not be compared because reverse distributor pickup occurred before the start of the preimplementation period; however, facility 2 demonstrated a reduction in total reverse distributor costs and cost per item (Table 6). Facility 1 saw an increase in the cost of expired CS doses, while facility 2 saw a reduction. Both facilities had a reduction in the cost of unused CS doses.

**Table 6.** Reverse Distributor Costs and Costs Tied to Expired and Unused Controlled Substances

Facility	Total reverse distributor cost^a^		Reverse distributor cost per item		Total cost of expired CS doses^a^		Total cost of unused CS doses^a^	
	Before	After	Before	After	Before	After	Before	After
1	NA	$8,965.09	NA	$5.37	$1,857.77	$7,015.37	$26,513.01	$3,780.05
2	$1,633.14	$126.37	$1.77	$0.58	$1,408.75	$678.43	$4,660.38	$1,415.33

Abbreviations: CS, controlled substance; NA, not applicable.

^a^Data collection for the preimplementation period took place over 30 days (March 31 to April 29, 2022), while data collection for the postimplementation period took place over 91 days (July 5 to October 3, 2023). To achieve comparability between data points, estimates from the postimplementation period were divided by a factor of 3.03 (91/30) and rounded to the nearest whole number.

#### Direct labor costs

Despite a reduction in overall direct labor costs, the low volume of logged entries in the postimplementation period at both facilities prohibited accurate measurement of impact.

### Module 3

***Perception survey*.** Before the intervention, 4 staff members answered a survey of 26 to whom the survey was distributed (15% response rate) about their work practices with ADCs and the CS vault. Three respondents agreed or strongly agreed that they spent too much time on CS vault count corrections and too much time running and analyzing the surveillance report. All respondents agreed or strongly agreed that storing too many CSs led to waste due to expiry.

After implementation of the centralized server and software, 13 complete responses of 57 (22% response rate) to the postimplementation survey were recorded. Of these respondents, 54% (7/13 respondents) agreed or strongly agreed that they had fewer CS vault count corrections and 57% (8/14 respondents) agreed or strongly agreed that it takes less time to run and analyze the surveillance report and that they have less waste due to expiry.

## Discussion

Inventory management is an integral process for pharmacy departments to ensure efficient and cost-effective operations.^15^ This 2-phase study demonstrated that use of an ECS management system and inventory analytics software can decrease labor requirements and time spent on inventory management.

To maximize health-system resources, the amount of inventory should be tightly controlled, without excessive stock leading to inflated carrying costs. On the basis of survey results, both facilities forecasted that labor costs associated with managing discrepancies were reduced. Seven of 13 respondents agreed or strongly agreed that they had fewer discrepancies and CS vault corrections. After implementation of the new systems, both facilities saw improved inventory turns. This may signify tighter inventory control and better movement of CS inventory from delivery to storage in the central CS vault, to distribution to the unit, and finally for administration to the patient. Both facilities had better overall inventory control and better medication availability, as evidenced by fewer stockouts.

Savings related to labor efficiencies were demonstrated by the reduced time to receive and store medications, allowing staff to reallocate this time to direct patient care activities and other value-added activities. The number of transactions requiring intervention or further action such as a restock decreased, thereby making the workflow more efficient.

Time saved may also be attributed to more accurate reports, as less time was spent investigating discrepancies. Despite an increase in the number of transactions with associated discrepancies, less time was spent on average managing each transaction. This was due to the added functionality of the enterprise system, which provides more visibility to information by combining reports in a single view, compared to previous systems that required comparison of 2 or more reports to triangulate the steps leading to a discrepancy.

The reduction in the cost of unused doses at both facilities demonstrates improvement in inventory turns and management of CS stock on hand due to improved PAR level management. It should be noted that the average time per transaction was high compared to that for patient-specific vends because transactions often involved stocking the ADCs and multiple medications were withdrawn per transaction rather than medications being withdrawn individually. Before implementation of the ECS solution, patient-specific vends required manual data entry for various identifiers (eg, patient name, order number, date of birth). In the preimplementation period, facility 1 entered all fields within the safe for this workflow, while facility 2 did not. Additionally, an increase in total costs for expired CSs was seen at facility 1, which was primarily due to a single high-cost drug that is infrequently used but required stock based on the types of clinical procedures performed. If this one high-cost item were removed, a large reduction in the cost of expired medications would be expected.

Before implementation of the new technology, the health system had an inventory optimization tool; however, sites did not leverage it for management of CS inventory through the vault, as optimization was more manual in previous CS vault versions. Utilization of the new system streamlined the workflow by eliminating the need to go to the CS vault and manually run a report and perform calculations, instead allowing changes to be made directly through the optimization tool. At baseline, invoices were manually reconciled. Although the time metric associated with this was not tracked in either the pre- or postimplementation period, due to the nature of manual data collection, staff perceived that there was a reduction in the time spent on this activity due to the functionality of pulling information from multiple sources into a single report electronically.

### Limitations

There are always challenges to the implementation of new technologies; during the limited commercial release of this newly developed solution, the adoption of products was postponed for approximately 12 months while some related technical gaps were addressed. Specifically, the first version of the software did not accommodate multiple National Drug Code numbers during an invoice receiving process and reporting was not specific to single facilities within the IDN. As such, a software update was required to accommodate preferred workflow. Even with this delay, the adoption period was kept to 91 days to allow for adjustments to the new software, which was true to the study design.

Despite the advantages seen with integration of technologies aimed to improve CS management, several limitations were identified. One of the main limitations in this study was the use of manual logs for data collection. This may have led to an underrepresentation of actual activities and transactions; time spent per transaction was used as a metric to ensure consistency. Time for activity logs was recorded in hours and minutes, and, as such, activities taking less than 60 seconds were rounded to zero minutes. Specific types of activities were inconsistently reported in the manual logs in the pre- and postimplementation periods of the study. The addition of new users to the database was also infrequently logged and could also have led to inconsistencies in the data analyzed. Given the length of time between the data collection periods, the authors believe that staff knowledge of elements required to be logged may have been overlooked. However, the authors believe that the challenges seen with manual logging reflect real-world practices.

An additional limitation was the lack of data on PAR level changes at baseline, making it difficult to determine the impact of the ECS solution. Establishment of PAR levels by predictive analytics was integrated into the workflow in the postimplementation period and helped improve the process of making ordering decisions. The new connectivity of the CS vault with the inventory optimization system allowed users to see daily use and adjust PAR levels based on these data rather than depending on a manual calculation or guessing. The functionality enhanced visibility of current stock, allowing staff to accurately determine exact stock levels and make precise decisions based on the data. Additionally, as a larger number of surveys were sent in the postimplementation period, there may have been a reduction in the ability to fully assess the perceptions of pharmacy staff on the impact of the ECS solution.

Of the 2 facilities, facility 2 saw greater process changes between the pre- and postimplementation periods; switching from a manual ordering process to CSOS increased some efficiencies, resulting in increased ordering frequency, although it is unclear how this impacted the results of the study. Facility 2 still saw improvements in most of the study endpoints.

Additionally, the timeframe of the study, from 2022 to 2023, coincided with a stabilization of the workforce as the technician shortage was addressed at the 2 facilities. In 2022, both facilities had a high vacancy rate for pharmacy technicians and surges in COVID-19 admissions. These challenges were seen at a lower frequency during the postimplementation period.

Lastly, drug shortages during the study period also required changes to ordering practices to ensure continuity of patient care. During the study period, the ECS solution accommodated only primary wholesaler data feeds in the receiving workflow at the vault. During the study, ordering from multiple wholesalers and direct shipments occurred, which required manual entry of additional wholesaler data. It is recommended that future solutions accommodate multiple data feeds to decrease manual data entry and further close the loop that can exist in the ordering and receiving process. The limitations of this research highlight the complexity of CS management as well as optimization of new technology in healthcare settings.

## Conclusion

A CS inventory management system integrated with an analytics tool provided a reduction in the amount of time spent on receiving, storing, and reconciling CS records even as the number of transactions increased. This provided increased visibility to gaps in the chain of custody for CSs and better inventory management, including fewer stockouts, higher turn rates, and a decrease in the number of expired medications. Integration helped close the loop between ordering and receiving inventory and eliminated manual processes. Future research studies should focus on the impact of tighter CS inventory management on the risks for drug diversion.

## Supplementary Material

zxae305_suppl_Supplementary_Table

## Data Availability

The data underlying this article will be shared on reasonable request to the corresponding author.
