# Supplementary material for: Impact of an enterprise controlled substance management system on labor and inventory costs
Source: Am J Health Syst Pharm. 2024 Oct 26;82(8):427–34. doi: 10.1093/ajhp/zxae305 (PMC12003853; doi:10.1093/ajhp/zxae305)
Supplement: zxae305_suppl_Supplementary_Table [file zxae305_suppl_supplementary_table.docx]

| **Terms** | **Definitions** |
| --- | --- |
| Discrepancy | When the expected and actual counts of a medication are not equal **and** the product is missing |
| Variance | When the expected and actual counts of a medication are not equal |
| Surveillance report | Report that documents variances and discrepancies generated by CS safe that may include variances between items sent from the safe and the automated dispensing cabinets. |
| Time managing and reconciling the surveillance report | Total time spent reviewing and reconciling the discrepancies generated by users on the surveillance report |
| CS vends | Vends or dispenses from the CS safe to the ADCs on patient floors and patient specific vends |
| Patient specific vends | Vends or dispenses from the CS safe for individual patients |
| CS stockout percentage | Number of CS safe stockouts (when count was zero) divided by the number of CS vends |
| Cost of expired doses | The cost associated with CS that expired during the data collection period |
| Cost of unused doses | The cost associated with CS that were never dispensed during the data collection period |
| Inventory turns | Inventory turns are a measurement of inventory efficiency that looks at the number of times your inventory turns over in a given period. This was calculated for the CS safe using the starting inventory, the ending inventory, and the list of purchases during the study period. |
| PAR level changes | Total number of PAR level (minimum and maximum quantity limits) changes in the controlled substance safe |
| Reverse distributor | A company registered with the DEA who collects controlled substances from sites registered with the DEA to either return them to the drug manufacturer or to aid in their disposal when needed. |

**eTable 1.** Definitions

Definitions: ADC, automated dispensing cabinets; CS, controlled substances; DEA, Drug Enforcement Agency; PAR, periodic automatic replenishment
